# Simulated real-world feasibility and feedback session for a lift assistance device, Raymex™: a mixed-method descriptive study

**DOI:** 10.3389/fresc.2024.1455384

**Published:** 2024-09-06

**Authors:** Michael Kalu, Andrew Chaston, Niousha Alizadehsaravi, Mirella Veras, Caitlin McArthur

**Affiliations:** ^1^ School of Kinesiology & Health Science, York University, Toronto, ON, Canada; ^2^ School of Physiotherapy, Dalhousie University, Halifax, NS, Canada; ^3^ Department of Physical Therapy, College of Rehabilitation Sciences, University of Manitoba, Winnipeg, MB, Canada

**Keywords:** fall risk, lift assistance, aging, medical device, mobility

## Abstract

**Background:**

Fall risk and incidence increase with age, creating significant physical and mental burden for the individual and their care provider. Lift assistive devices are used in multiple healthcare facilities, but are generally not portable nor self-operational, limiting their use outside of medical supervision. The Raymex™ lift is a novel lift assistance device within a rollator to address these limitations. We aim to gather user-centered feedback on the Raymex™ lift, set up instructions, safety protocols to improve feasibility and usability, and explore the potential usability as a fall recovery or prevention device.

**Methods:**

Four older adults, two informal caregivers and 16 formal caregivers (clinicians and continuing care assistants) participated in a focus group. Participants provided feedback on the Raymex™ lift after viewing a demonstration and using the device. Qualitative and quantitative data were analysized using thematic and descriptive analysis respectively.

**Results:**

Participants highlighted three major themes: (1) Design features requiring improvement, (2) Positive feedback and suggestions to optimize the Raymex™ lift and (3) Pricing vs. social utility. Participants suggested widening the seat, changing the braking button layout, and lowering the device weight to improve usability. Participants believed the main device feature was fall recovery and had implications for social utility by reducing the need for ambulance visits to the home. Price point led to a concern on affordability for older adults.

**Conclusion:**

The feedback gained will advance the development of the Raymex™ lift and may highlight cost-effective design choices for other developers creating related aging assistive technologies.

## 1 Introduction

**Figure 1:**
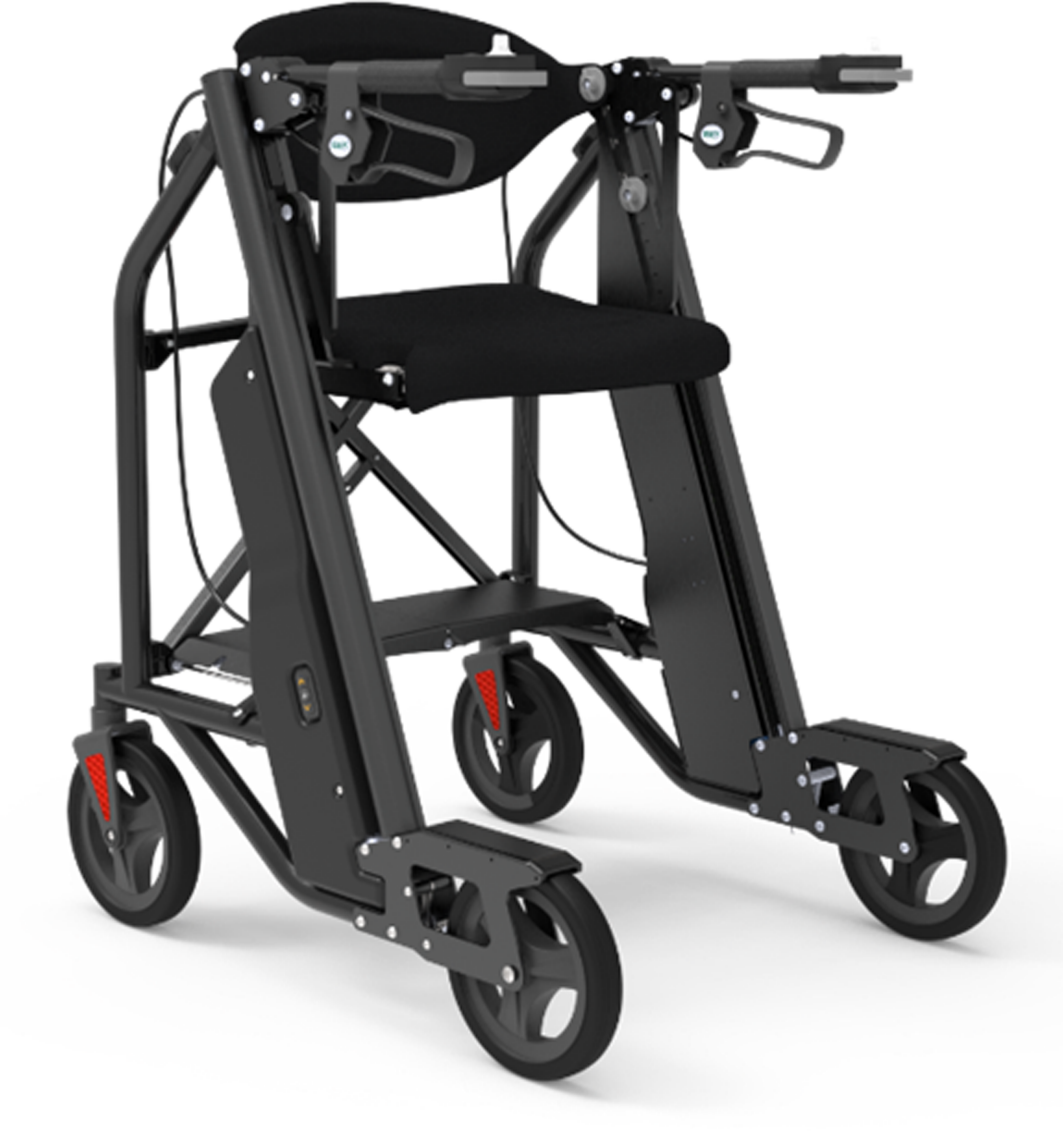
Image of the Raymex™ lift (V4) prototype.

With aging, there is an increased falls risk due to age-related physical changes in balance and gait, neuromuscular changes in muscle mass, strength, and power, and changes in cognition (1, 2). The global prevalence of falls among older adults is significant, ranging from 26% to 35% overall and 32% to 42% for those aged 70 older, (3, 4) and costing $2 billion and $50 billion respectively, (5–7) with each fall costing $2,044 to $6,606 for those aged 65 years or older (8, 9). Non-fatal falls also contribute to ongoing healthcare costs, with ambulance visit costing at least $224 in the United States, and between $45 to $325 in Canada, depending on province (10–19). The reliance on emergency services for fall-related injuries further increases the burden on the caregiver both physically and mentally (20–23).

(5–7) Due to the large physical burden on caregivers, emergency response wait-times, and healthcare associated costs with lifting older adults after a fall, many companies have attempted solutions with fall assistance devices, for example The Hoyer elevate, (24) IndeeLift Human Floor Lift for Fall Recovery, (25) or Bellavita Dive Bath Lift (26). While some of these lifts have helped older adults from the floor level, there continues to be shortfalls including the lifts’ weight, requirement of an additional person to operate the device, low maximum seat height, and non-motorized seat height adjustment (24). To fill this gap, *Axtion Independence Mobility Inc.* has developed the Raymex™ lift (https://raymexlift.com), a mobility aid device with a similar structure to a rollator walker, but with the added feature of lift assistance and recovery up to a maximum weight of 300lbs (Figure 1).

The Raymex™ has a battery powered movable seat that can descend to floor level, rise to a height of 24” and stop anywhere in between. It can be operated by the older adult on the floor via a control panel on the bottom panel of the structure, while they are on the seat using the control panels on the handles, or by a caregiver via a control panel on the top of the frame. While scholarly literature supporting existing or similar lifts to the Raymex™ is limited, previous evidence has reported that physical demand is much higher in floor lifts compared to robotic-assisted transfer lifts, and users reported discomfort during use (27, 28).

After multiple design revisions including features and structural engineering, *Axtion Independence Mobility Inc* is approaching a market ready product (Class 1 Medical Devices) (29). Users feedback on the Raymex™ will enable final modifications to prepare the final market ready device. This mixed-method study has two objectives, to: (a) gather feedback on the Raymex™(V4), and users set up instructions and safety protocols from older adults’, informal caregivers’, formal caregivers’ (e.g., personal support workers), and clinicians’; and (b) explore the potential use of the Raymex™ as a fall recovery or prevention equipment.

## 2 Methods

A mixed-method descriptive study (30) was used to seek feedback from older adults, informal and formal caregivers about the comfort, usability, and design of the Raymex™. The qualitative and quantitative data were mixed within the result and discussion sections.

### 2.1 Ethical approval

This study was reviewed and approved by the Dalhousie University Health Science Research Ethics Board (REB: 2023-666).

### 2.2 Participants and sampling

Four participant groups were gathered using criterion-based purposive sampling (31): older adults residing in independent living or their own home; informal caregivers (e.g., family or friends); formal caregivers (e.g., paid personal support workers); and clinicians (physiotherapists, occupational therapists, and their assistants). Inclusion criteria for older adults were: aged 65+, self-reported recent fall or fall risk or fear of falling, lives alone or with someone, uses walking aid, speaks and understands English. Exclusion criteria included recent surgery (past six months) or injury on the lower limb, comorbidities preventing participation, and cognitive impairment preventing comprehension. Informal caregivers were eligible if they had provided at least two months of unpaid care to an older adult meeting the study's inclusion criteria while formal caregivers and clinicians have experience working (at least 6 months) with older adults that met the inclusion criteria. Sample size recommendations for feasibility mixed method descriptive study depend on the purpose of the study and the weight of the qualitative or quantitative components (32, 33). Our study does not aim to generalize our findings; hence we weigh the qualitative components higher and that guided our sample size. The sample size for qualitative description studies (34) has been based on data sufficiency or saturation—where no new theme emerges during interviews (35). Based on this, we estimated a sample size of 20 participants (five from each group) to allow for comprehensive feedback on the Raymex™, achieving data sufficiency or saturation.

### 2.3 Recruitment

Older adults and their family members were recruited from a healthcare organization offering independent living and home care services, while clinicians were recruited from a rehabilitation clinicians special interest group working in long-term care (LTC) in Nova Scotia. Recruitments involved study flyers on the independent living buildings' notice boards and in their weekly newsletters, and targeted emails to the special interest groups detailing research objective and researchers' contact information. Interested participants contacted the researchers, underwent screening and, if eligible, were invited to take part in a focus group discussion.

### 2.4 Data collection

Data were collected over three sessions, each lasting 2 h, from November 2023 to January 2024. The sessions were a mix of in-person and hybrid and were held separately for older adults/informal caregivers; physiotherapists, occupational therapists, and physical/occupational therapist assistants; and support care workers. At each session, a minimum of four researchers and one Raymex™ developer (product engineer or the chief executive officer) were present. During the focus group discussions, participants watched an in-person demonstration of the Raymex™. They practiced using the Raymex™ as guided by licensed physiotherapists. Participants were video recorded while they provided ongoing feedback during the practice sessions. Following the practice, participants completed the questionnaire and submitted their feedback on the user setup instructions manual and safety protocols. Participants who were unable to provide written feedback on the documents provided oral feedback. Each session ended with a focus group discussion guided by semi-structured questions focused on usability (e.g., ease to use), comfort, safety, perceived impact, and physical experience of this device (see Appendix 1 for the focus group guide). Field notes were taken by research assistants at each session and informed the context during data analysis. Audio data was recorded and transcribed using otter.ai (Otter.ai, Inc., Mountain View CA), an artificial intelligent transcription services, and was check for correctness by research assistants.

### 2.5 Data analysis

The qualitative data derived from transcripts and field notes were analyzed by the research team employing directed thematic content analysis (36) managed in NVivo 1.6.1 (Lumivero, Denver, CO). Four coders independently coded the data to identify major themes, including areas for improvement for the Raymex™. All coders met to merge their coding. De-identified videos were also analyzed using an open-coding principles to identify participant interactions and movements related to the practice of the Raymex™, including any practice problems not captured verbally in the focus groups. Themes derived from field notes, transcripts of focus group discussions, and video analyses were triangulated and discussed in meetings to address and resolve any differences in interpretation and/or coding.

Quantitative data, including demographic information and results from the survey, were managed in Microsoft Excel, and are presented in aggregated format. Descriptive statistics, such as frequency, percentages, mean, standard deviation, were used to describe the quantitative data.

### 2.6 Trustworthiness

We employed member checking by sending the study's findings to the participants to ensure that we captured all their feedback and ideas regarding the Raymex™ (37). We triangulated qualitative and quantitative survey data enhancing the credibility and kept an audit trail of the research process, ensuring transparency, and facilitating future verification. We kept reflective notes which described the Subjective I—those values, assumptions, and beliefs that a researcher brings to research. For instance, three authors are physiotherapists and they reflected on how their profession as physiotherapist would influence how the feedback provided by the participants were interpreted. To enhance the dependability of the study findings, multiple coders coded the transcripts and met to discuss the emerging themes.

## 3 Results

A total of 22 individuals participated in three focus groups. Four older adults participated, aged 73–85 years [mean (SD) = 79.25 (5.68 yrs)], two female and two males, had some education (e.g., a diploma and bachelor's degree), two lived alone in independent living buildings, and two resided at home or in assisted living with their caregivers, and three had a walking aid (either a rollator, a stick or both). Two informal caregivers participated who were aged 69 and 75 years old [mean (SD) = 72(4.24 yrs)], a male and a female, with bachelor's and high school education. Clinicians (eight physiotherapists, one occupational therapist, and four physiotherapy/occupational therapy assistants) and formal caregivers (three continuing care aides) included 12 females and four males, all full-time staff working in a LTC setting aged 27–64 years [mean (SD) = 44.08 (12.51 yrs)]. One physiotherapist did not report their age.

### 3.1 Themes

Three overarching themes with several subthemes emerged as feedback regarding the real-time use of the Raymex™ (Table 1): (a) Design feature requiring improvement; (b) Positive features and suggestions to optimize the Raymex™ features and usage; and (c) Pricing vs. social utility. Theme development is shown in Appendix 2.

**Table 1:** Themes and subthemes of feedback regarding the real-time use of Raymex™.

Theme	Subthemes	Quotes
Design feature requiring improvement	• Design-related to confusion of some Raymex™ lift features • Design-related to features of Raymex™ lift • Design-related to Raymex™ lift safety	*1a) “One thing I noticed is its quite loud. The motors I mean, it's not a huge deal, but is there a way to soften that?” (clinician)* *1b) “Because I found it difficult to fold (with string attachment); I also found getting the seat up the handle that piece of string you out there? I understand you're gonna have an actual lift there. because I found it difficult to fold it.” (older adult)* *1c) “Yeah. I felt a bit uncomfortable” (Older adults)* *1d) “… it's not safe to set on and move around with that. It seems that it is not steady but move forward and backwards.” (clinician)* *1e) “The push buttons and stuff like to go up and down. I think not on the up and down. The braking is a little bit confusing. Like the brake button. I think that's something you would get used to pretty quickly but it's a little confusing at first.” (informal caregiver)* *1f) “Yeah, the automatic aspect kind of you’re not sure whether it's locking or not locking.” (informal caregiver)*
Positive feedback and suggestions to optimize the Raymex™ lift	• The synergy of design-positive features in the Raymex™ lift enhancing usage • Suggestion to optimize Raymex™ lift features and usage	*2a) “So, it sounds like the kind of main feature that's unique and you feel comfortable is the ability to get up off the floor.”* *(older adult)* *2b) “In places where there are no grab bars, which is most places, this would be helpful. I'm not sure of things, grab bars, you know, do prevent falls. But if they're not there or they're in the wrong place, then something like this would be (more) helpful than your regular walker.” (informal caregiver)* *2c) “Somebody fell in our parking garage in our building on a Sunday evening. And we called and they said we are really short on ambulances right now. People call in but an hour later, he's still lying on the floor of the garage—Raymex™ would have been useful here— however, And I finally went out on the street. There are a couple of people coming from the hockey game, strong people and they came to help me get him up” (informal caregiver)* *2d) “Another thing that feels good about the weight is that it feels so much more solid than the lightweight (older adult)”* *2e) “Walker that it would be more helpful than the regular walker”. (older adult)* *2f) “So, water resistant, not waterproof, can't take it and shower with it. But it's water resistant. So, if you're out in the rain and the splashes are coming up, you're okay.” (clinician)*. *2 g) “This (backrest) seems to need to be bigger or something.” (older adult)*. *2 h) “When you're like in a position like this where you're a bit lower, can you propel? (clinician)* *2i) “What about the battery if you're there, what does its normal user look like? And what if I'm someone going up and down and up and down?” (clinician)* *2j) “How do you clean the seat if its soiled? (clinician); Are all the parts able to be replaced or purchased of worn out? (clinician)”* *2k) “Is there a brake adjustment, because with that type of cable system, even that they do typically loosen over time, and it looks like it's still got that same type of screw.” (clinician)*
Pricing vs. social utility	• Pricing consideration is essential • Social utility—potential societal impact of adopting the Raymex™ lift • Potential to save costs for ambulance services	*3a) “I like it. but, for three grand I had to cut my tub down to get in and out for shower here and that cost me $2,000. I don't mind spending the money. I think it's a great piece of equipment. But I think your pricing has to be competitive. And I'm not suggesting that you have to come down to $500. But I think you certainly going to have a marketing problem selling that at $3,000”. (older adult)* *3b) “Do you think it would be covered by private insurance, they cover walkers and wheelchairs but the costs between those two are very different (clinician); Does insurance cover this?” (older adult)* *3c) “Do you think it could be covered by private insurance they cover walkers and wheelchairs but the costs between those two are very different?” (clinician)*

#### 3.1.1 Theme 1 – design feature requiring improvement

This theme highlights design-related elements requiring improvement, such as noise of the seat raising and lowering and safety. Our participants highlighted their concern regarding noise from the Raymex™ motor, especially if multiple older adults use it in the same home.

Another concern elucidated by our participants is the substantial weight of the Raymex™, which may impede its outdoor use, particularly for caregivers or older adults who may be frail. Participants expressed varying perceptions of the use of the Raymex™ across different environments: 87% found it useful in their current residence, 82% for home care services and 68% the Raymex™ in LTC settings, but only 52% found it useful for community travel.

Both clinicians and older adults voiced safety concerns during the real-time use of the Raymex™ emphasizing the difference in stability when seated and in motion. Participants inquired about seat responsiveness to obstacles when lifting older adults and generally shared sentiments of discomfort and unease, underscoring the importance of heightened stability to prevent re-fall from the lift.

Participants also commented on the narrow width of the seat expressing concerns that it could limit the comfort and mobility of users with different body shapes. An older adult also expressed concern regarding the comfortability of the Raymex™ related to the edge of the seat pressing into the back of the legs. The participant was not alone in their concern as 3 participants (∼15%) reported the seat to be uncomfortable on the questionnaire.

The feedback from an informal caregiver revealed notable observations on the usability of the Raymex™, explicitly concerning the push buttons for ascending and descending. The caregiver highlighted initial confusion, particularly regarding the brake button, expressing the belief that familiarity would develop over time. However, this may have been an isolated incident as only one participant (∼5%) noted they did not like the raising/lowering button design. Another informal caregiver echoed this sentiment, emphasizing uncertainty regarding the automatic braking mechanism, indicating a potential challenge in discerning whether the mechanism is engaging the brakes. This was supported by three participants (∼15%) reporting they did not like the braking button design on the questionnaire. When probed to suggest ways to improve the button layout, three participants (∼15%) mentioned they would like the braking button to be separated into a unique locking and unlocking button, one participant (∼5%) suggested a longer light interval to indicate locking or unlocking the brakes, and one participant (∼5%) suggested to use different button shapes to indicate the locking/unlocking function for those with visual impairments. These insights underscore usability concerns, specifically about the confusing nature of the brake button and the automatic feature.

#### 3.1.2 Theme 2: positive features and suggestions to optimise Raymex™

This theme encompasses a series of positive features and suggestions that would improve usage of the Raymex™. As highlighted by older adults, the Raymex™'s most significant feature is its unique and comfortable ability to facilitate getting up from the floor. The informal caregivers further emphasized the device's utility in locations without grab bars, noting its superior assistance to a regular walker. Participants further enumerated that these features promote usage, including its potential role in preventing falls, fall recovery, facilitating efficient assistance when needed (with or without a caregiver present), as well as offering a stable and solid support system. One informal caregiver provided a scenario where prompt assistance was required, and Raymex™ would have been helpful, highlighting the potential Raymex™ role in emergencies. On the questionnaire, participants expressed interest in additional features for the Raymex™. See Table 2 for the questionnaire results with quotes from the focus group supporting their suggestions.

**Table 2:** Survey questionnaire feedback on potential Raymex™ additional features.

Raymex™ feature	Participants’ supporting quotations from focus groups	Participants showing interest (%) on survey
Larger basket to hold larger items	*If a person is going to use it outside of the home, a basket on the back, or some type of carrier just for taking some small goods; Can it be used outside (clinician)*	100
Cup holder		86
Small pouch to hold your mobile phone or other small items	*If a person is going to use it outside of the home, a basket on the back, or some type of carrier just for taking some small goods. I'm not suggesting you go shopping in there, but it would be nice to have easy access to a cell phone in case you got into trouble and needed to call.” (older adult)*	82
Curb climbing attachment		65
Portable oxygen tank holder		64
Ability to use the Raymex™ as a powered transport the user controls		55
Cane holder		55
Ability to use the Raymex™ as a manual transport wheelchair pushed by another person		52
Ability to summon the Raymex™ to come to you by pressing a portable button	*“This is getting off the floors is an emergency situation. Have you thought of incorporating an emergency alarm on it?” (clinician)*	52
Umbrella holder		45
A Seat that can tilt forwards by a button push to assist standing up		30

Participants provided feedback on potential situations the Raymex™ may be useful for, and feedback on design (see Table 3). Participants believed the Raymex™ would be useful for (1) retrieving items from a low level/ground (78%, *n* = 18), (2) transferring from/to a seat (78%, *n* = 18), car (74%, *n* = 17), bed (70%, *n* = 16), toilet (78%, *n* = 18), (3) standing close to the sink (87%, *n* = 20), (4) sitting at counter height (83%, *n* = 19), (5) walking inside (70%, *n* = 16) and outside (61%, *n* = 14) the house (6) independent (78%, *n* = 18) and assisted (91%, *n* = 21) fall recovery, and (7) as an exercise tool (78%, *n* = 18). During the focus groups, clinicians inquired about durability aspects such as waterproofing and maintenance and address practical concerns regarding battery usage/performance during frequent up and down movements. Clinicians raised concern about the Raymex™'s maneuverability in a lowered position, distinguishing it from a wheelchair. They also inquired about practical aspects such as cleaning the seat, parts replacement, and brake adjustments.

**Table 3:** Example of the Raymex™ developers user guide, the suggested revision, and the final version.

Raymex™ developers user guide (original)	Revision suggestions	Final version
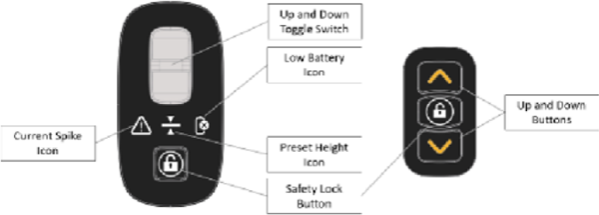	“Which switch is which? Is the left or right Upper/lower?”	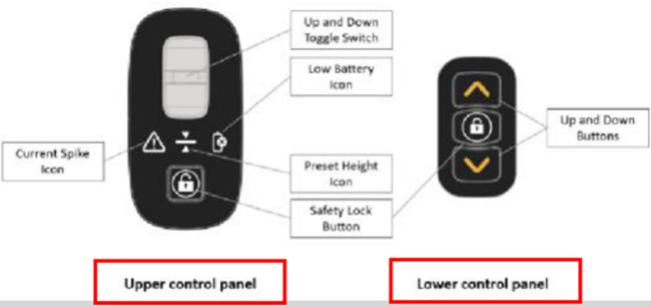
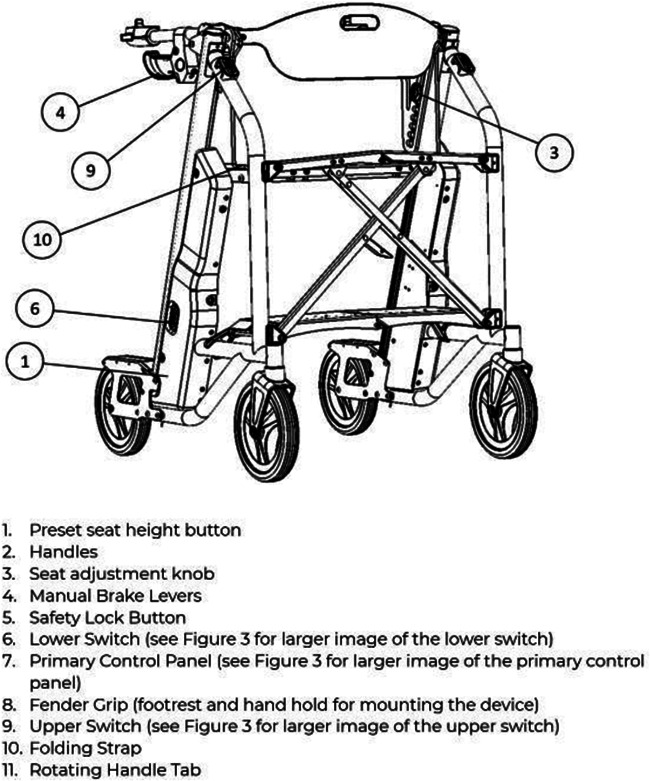 A second person (e.g., technician, clinician, companion, caregiver, etc.) should remove the clip-on side panel on the lower right side of the device (when facing the direction of travel). Locate the square button below the battery. Depress and hold the button for five (5) seconds until the Preset Height Icon on the Primary Control Panel (see Figure 3: The Raymex™—Primary Control Panel) flashes BLUE. The other icons will also flash their respective colours.	“This (access to the battery) should be labeled on the picture”	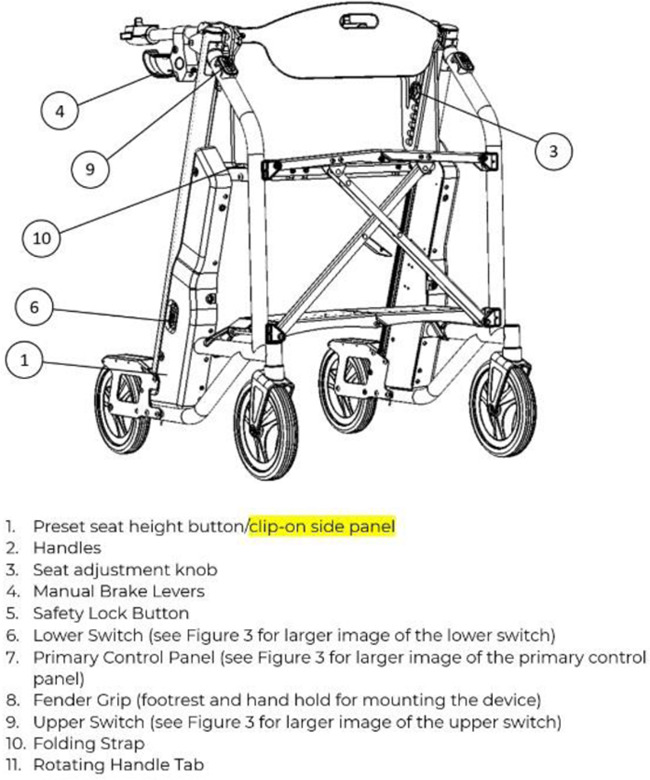
• Safety Lock: When the lifting seat is actuated by any of the controls, a separate electric parking brake will be engaged. Users can disengage the brake by pushing the Safety Lock Button on the Primary Control Panel which will flash GREEN (see Figure 3: Primary Control Panel). To engage the safety lock without using the lifting seat, press the Safety Lock Button and it will flash RED. • Turning: Engage the brake slightly on the same side of the desired turn direction. Turn your body towards the desire direction and then turn the walker towards in the same direction. Take small, controlled steps to complete the turn.	“Repetitive”	Safety Lock: • The Safety Lock works like a parking brake and can be engaged in three places: the Primary Control Panel, the Lower Switch, and the Upper Switch. • To lock the wheels, press the Safety Lock Button on the Primary Control Panel or the Upper Switch. The light should flash RED; if it flashes GREEN, press the button again. • To unlock, press the Safety Lock Button on the Primary Control Panel, Upper Switch, or Lower Switch. The light should flash GREEN; if it flashes RED, press the button again. • Note: The Safety Lock engages automatically when the seat is raised or lowered. Manual Brakes: • To slow down the Raymex™ while walking, pull up or squeeze the Manual Brake Levers. • Release the Manual Brake Levers to resume normal walking pace. • To stop and lock the wheels, push down on the Manual Brake Levers until they lock. To release, pull up on the Manual Brake Levers.
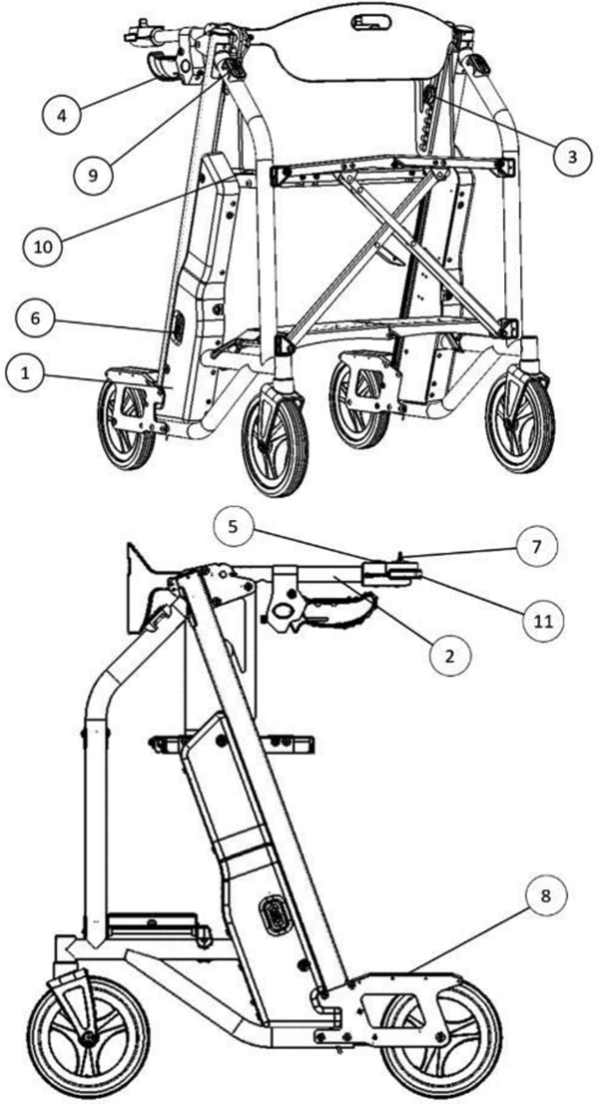	“Better continued numbering on the figures”	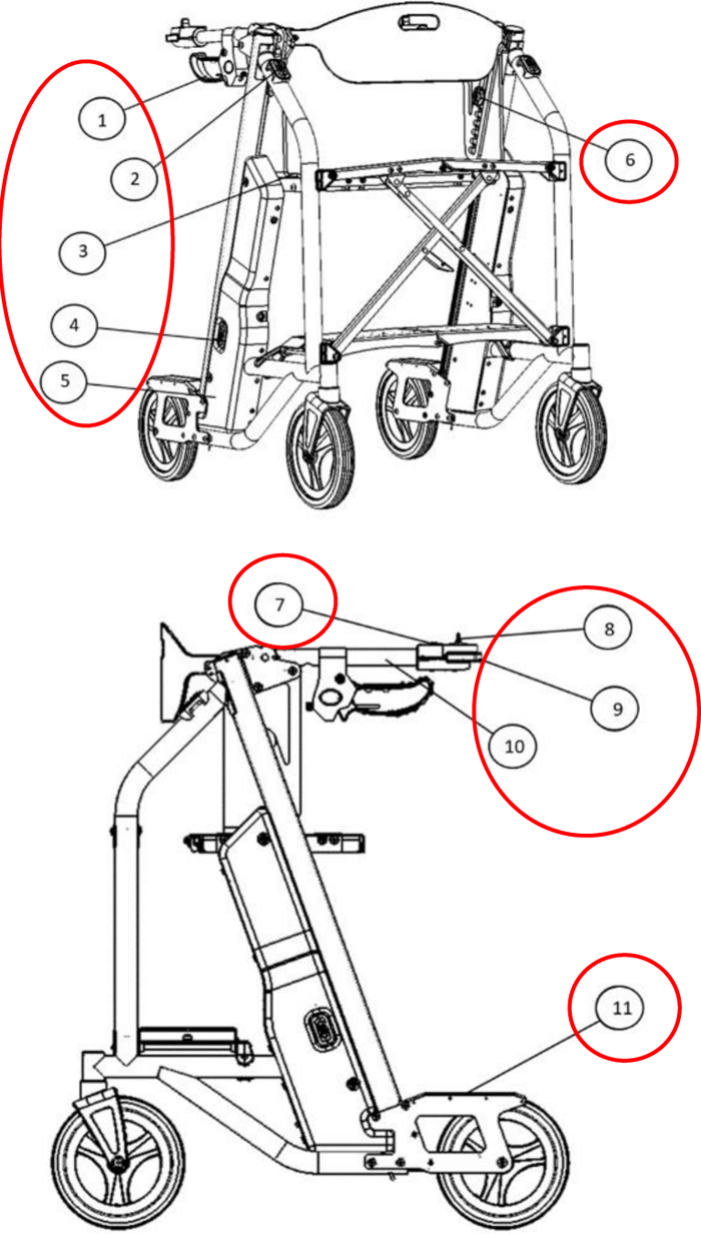

#### 3.1.3 Theme 3 – pricing vs. social utility

Participants frequently discussed the idea of pricing vs. social utility in the focus group. Competitive pricing strategies were discussed, ensuring that unit pricing aligns with the targeted demographic. Despite concerns about pricing and insurance coverage, participants emphasized the social utility of the Raymex™, citing its potential ability to meet both personal and societal needs. Participants highlighted potential cost savings, not only in terms of competitive pricing but also in the context of emergency services and personal expenses, envisioning a scenario where the Raymex™ could potentially save costs on ambulance services and reduce expenditures associated with hospital visits. According to the participants, the lift's social utility would outweigh any potential pricing issues, emphasizing the need for the government to invest in subsidizing the price. Participants also raised questions about insurance coverage, drawing comparisons with coverage for walkers and wheelchairs.

## 4 Discussion

The Raymex™ is a portable personal lift, providing an enhanced alternative to a rollator walker, featuring an elevating seat capable of descending to floor level and ascending to a height of 24 inches, with the ability to stop at any chosen height. The Raymex™ lift distinguishes itself from other existing lifts, such as the Indeelift Human floor lift and the Bellavita Dive Bath Lift, (26) due to its portability and ascending height of 24 inches, as opposed to 21 inches and 18.89 inches, respectively. This design may be particularly appealing to older adults and their family members due to its self-operational nature that is not present in FGA-700 lift (38). This study explored the usability of version 4 of the Raymex™ among older adults, informal caregivers, and clinicians, to elicit specific feedback regarding use, features, and instructional manuals. Our participants highlighted that Raymex™'s multi-purpose use, including its ability to facilitate getting up from the floor, and adaptability in spaces where other large lifts or walkers may not be practical. In addition, our participants provided feedback regarding some of the features of the Raymex™, mainly on the confusion of the brake button and the seat width. The participants asked additional probing questions with suggestions to enhance Raymex™ features and discussed its potential for social utility and pricing considerations.

Participants—primarily older adults and informal caregivers—have proposed supplementary features, such as a basket for holding phones and decreasing the lift's weight to facilitate outdoor use. Such suggestions underscore a user-driven demand for improved functionality and adaptability in the device, (39) addressing the preferences of older adults with mobility limitations who seek versatile equipment for enhanced confidence and comfort outside their homes. The desire for weight reduction stems from recognizing that frail older adults or informal caregivers may face challenges lifting the Raymex™ in and out of a car. The reduction in weight of the Raymex™ has the potential to facilitate aging in place, (40, 41) preserving a sense of identity through independence and autonomy by enabling older adults and informal caregivers to conveniently transport the Raymex™ into the community for use and return home, mitigating the necessity for LTC as frailty increases.

Our participants shared experiences or concerns related to the affordability of the Raymex™, which was not surprising given the historical challenges associated with pricing considerations for innovative tools targeted at older adults (42). Previous work has reported the impact of pricing on adoption, focusing on affordability for market penetration, and accessibility for equitable and inclusive mobility aids (43, 44). Nevertheless, participants perceived the relationship between the pricing of the Raymex™ and the social utility as essential to improving the health and well-being of older adults/informal caregivers. Striking a balance between pricing and social utility is promising to support the desire of older adults to age at home and reduce ambulance-related costs for falls among older adults. Participants perceived that government adoption of the Raymex™ or developed policies to subsidize the cost could potentially lower current ambulance call expenses for recovery assistance, which range from $45 and $325 in Canada and the United States, respectively (17,18).

### 4.1 Limitations

While our study's strength is based on the real-time feedback of the Raymex™, there are some limitations. We have a relatively small sample of older adults and informal caregivers recruited from one healthcare organization in Nova Scotia resulting in feedback that may not be comprehensive and generalizable. In addition, we were not able to include all team members to provide feedback on the device such as nurses and geriatricians. Subsequently, future usability experience or input on the next version of the Raymex™ should incorporate a diverse population of older adults and clinicians. The presence of a product design engineer and the CEO of the company at the feedback sessions may have limited the negative feedback participants feel comfortable providing. Despite the limitations, feedback is essential for continuous improvement and enhancement of user-centred design and usability of novel devices like the Raymex™. Our results can be used by future developers of technologies to support aging in place and cost-effective design choices in early development. In our specific scenario, the Raymex™ developers will utilize these insights to formulate the final version of the Raymex™ for a pilot feasibility test, laying the groundwork for a conclusive trial to ascertain market readiness.

## 5 Conclusion

Our participants highlighted that the significant feature of the Raymex™ was its ability to lift older adults off the floor and act as a walker, and it can easily be adapted to different home settings. However, participants also highlighted improvement of some Raymex™ features, such as a narrow lift seat and button settings, which would enhance the lift's usability. Additional features were suggested, including reducing the weight of the lift to enable older adults and informal caregivers to use it outside the home. This comprehensive feedback not only provides valuable suggestions that can guide potential refinements and advancements in the Raymex™ design, but also emphasizes its potential to enhance mobility and safety of older adults. Our results can be used by future developers of technologies to support aging in place to support cost effective design choices in early development.

## Data Availability

The raw data supporting the conclusions of this article will be made available by the authors, without undue reservation.

## Appendix 1

**Interview guide for focus group discussion after the demonstration**

***Note: Most questions are general and those specific to a specific group is indicated in the guide, and highlighted in red.***

1. How do you feel when you practiced using the Raymex™ lift in terms of comfort and safety?

Probes

○ How straightforward was the setup process for the Raymex™ lift
○ Are there technical challenges you encountered when you were practicing using the Raymex™ lift

2. Were there any pressure points or discomfort that you experienced when you practiced the demonstration?
3. How easy or difficult was it for you when you practiced the demonstration?

Probes

○ Did you face any challenges or confusion while operating them?
○ Where the controls self-explanatory to understand?

4. What are your thoughts on how helpful the Raymex™ lift would be to older adults, their family caregivers, formal caregivers (e.g., personal support workers), and clinicians?

Probes

○ Aside from lifting someone off the ground and preventing falls, what are the other things this Raymex™ lift can be used for?
○ In your experience, how do you think patients, family caregivers, and clinicians would respond to the Raymex™ lift

5. Based on your experience as a clinician or caregivers (formal), what are your thoughts regarding using the Raymex™ lift as a fall rehabilitation or preventive tool?

Probes.

○ How can you use this the Raymex™ lift as a fall rehabilitation or preventive tool?
○ Are there specific features that may need modification, and how do you suggest we modify?

6. Based on your experience as a clinician or caregiver (formal), what are your ideas regarding integrating the Raymex™ lift in your care planning?
7. Based on your experience, do you have any recommendations for enhancing the Raymex™ lift usability and functionality?
8. Is there any other thing you would like to discuss regarding the demonstration and the practice of the Raymex™ lift?

**Interview guide for focus group discussions on the user setup and safety protocol.**

***Note: This guide will be used for all groups, however some questions are specific for a group and those are highlighted in red for the group***

**Users’ set-up draft**

1. How explicit and easy to understand were the user instructions for the Raymex™ lift
2. Were there any aspects of the user setup draft that you found confusing or unclear?

Probes:

○ Please can we discuss those areas in the document?
○ What are your suggestions to enhance clarity?

3. Did the instructions adequately guide or would guide you through the process of setting up the Raymex™ lift?

**Safety instruction**

1. Did you think that the safety instructions were comprehensive?
2. Were there any aspects of the safety instructions that you find confusing or unclear?

Probes:

○ Please can we discuss those areas in the document?
○ What are your suggestions to enhance clarity?

3. As an older adult, did the safety protocol provide you with a clear steps to ensure yourself when you are using Raymex™ lift?
  ○ Were there any safety measures you found that would be particularly crucial when using the Raymex™ lift and why?
  ○ Are there any other safety measures that we should consider—please describe them?
4. As a caregiver or clinicians, did the safety protocol provide you with clear steps to ensure the older adults’ safety when using the Raymex™ lift?

Probes:

○ Were there any safety measures you found that would be particularly crucial when using the Raymex™ lift and why?
○ Are there any other safety measures that we should consider—please describe them?

5. Communication is often an issue during emergencies, such as falls. As a caregiver or a clinician, how well did the Raymex™ lift design and safety protocol facilitate communication between you and the older adults?
6. As a caregiver or a clinician, did the safety protocol address common scenarios and concerns you might face when using the Raymex™ lift based on your caregiving experience? If not, what are the scenarios and concerns we should consider incorporating into the Raymex™ lift
7. Is there any other information regarding the user’s guide and safety protocol that you want to discuss?

## Appendix 2

**Table:**

Quotes	Codes	Category	Subtheme	Themes
One thing I noticed is it’s quite it's quite loud. The motors it I mean, it's not a huge deal, but is there a way to soften that? (clinician)	Loud noise motor	Design-question relating to noise pollution	Concern for noise related to the equipment	Design features requiring improvement
One thing I noticed is it’s quite it's quite loud. The motors it I mean, it's not a huge deal, but is there a way to soften that? (clinician)	Equipament noise	Design-question relating to noise pollution	Concern for noise related to the equipment	Design features requiring improvement
One thing i noticed is its quite loud. The motors i mean, its not a hugh deal, but is there a way to soften that? (clinician)	Loud	Design-questions regarding noise	Concern for noise related to the equipment	Design features requiring improvement
Because I found it difficult to fold (with string attachment); because I found it difficult to fold it.participant (?); I also found getting the seat up the handle that piece of string you out there? I understand you're gonna have an actual lift there. because I found it difficult to fold it. (older adult)	Difficult to fold	design-concern/difficulty	Design related to confusion of features	Design features requiring improvement
Yeah, the automatic aspect kind of you're not sure whether it’s locking or not locking. P2, informal caregiver	Locking and unlocking confusing	Design-confusion	Design related to confusion of features	Design features requiring improvement
The breaking is a little bit confusing. P2, informal caregiver	Break confusing	design-confusion	Design related to confusion of features	Design features requiring improvement
The push buttons and stuff like to go up and down. I think not on the up and down. The breaking is a little bit confusing. Like the break button. I think that’s something you would get used to pretty quickly but it's a little confusing at first. (informal caregiver)	Usability—Break button is confusing	design-confusion	Design related to confusion of features	Design features requiring improvement
Yeah, the automatic aspect kind of you're not sure whether it’s locking or not locking. (P2)	Confusion about automatic button	Design-confusion	Design related to confusion of features	Design features requiring improvement
The push buttons and stuff like to go up and down. I think not on the up and down. The breaking is a little bit confusing. Like the break button. I think that’s something you would get used to pretty quickly but it's a little confusing at first. (P2)	Confusing button, regarding breaking or not	Design-confusion, but easy to learn	Design related to confusion of features of Ibexlift(c)	Design features requiring improvement
I noticed that the seat is sharp and cuts into the back of your legs. Like the front edge. (older adult)	Comfort and safety—Front edge of the seat is sharp	Design-negatives	Design related to features of Ibexlift(c)	Design features requiring improvement
I felt a bit uncomfortable because as I said, at the time, the seat was kind of narrow. But whereas your device wasn't. (older adults)	Seat narrow	design-negatives	Design related to features of Ibexlift(c)	Design features requiring improvement
“felt a bit uncomfortable beacuse as I said, at the time, the seat was kind of narrow(P2 Older adults)”	Narrow seat	design-negatives	Design related to features of Ibexlift(c) -	Design features requiring improvement
Why is the device not movable? Is it because of the seat on the floor? (clinician); When you use the electronic brake, its not effecting the manual brake is it (clinician); Are all the parts able to be replaced or purchased of worn out? (clinician); Is there a brake adjustement, because with that type of cable syste, even that they do typically loosen over time and it looks like it’s still got that same type of screw (clinician); Are those long cables brake cables? (clinician)	Braking system	Design-device performance?		Design features requiring improvement
So class one is sort of the lowest risk device (clinician)	Low risk device	Design-low risk		Design features requiring improvement
feeling like I might fall off it or something (older adult)	safety—Sensation of sliding off the seat	Design-safety	Design related to Ibex lift safety	Design features requiring improvement
I also found getting the seat up the handle that piece of string you out there? I understand you're gonna have an actual lift there (older adult)	Safety issue	Design-safety	Design related to Ibex lift safety	Design features requiring improvement
is it like safe like other later it’s not safe to set on and like move around with that. When you say on you have to be still but for that one we can move forward and backwards. (clinician)	Safety concerns	Design-safety	Design related to Ibex lift safety	Design features requiring improvement
will the seat stop lowering if there’s an obstacle in the way like someone's put or cap? (clinician)	Safety concerns related to seat lowering	Design-safety	Design related to Ibex lift safety	Design features requiring improvement
You know a little uncomfortable feeling like I might fall off it or something (PI); Yeah. I felt a bit uncomfortable because as I said, at the time (P2; Older adults)	scared of falling because of the ibex lift	Design-safety	Design related to Ibex lift safety	Design features requiring improvement
You know a little uncomfortable feeling like I might fall off it or something (older, adult)	Comfort and safety—Feeling uncomfortable	design-safety & comfort	Design related to Ibex lift safety	Design features requiring improvement
So what type of people are you marketing? (P3, older adult)	“consideration for the target population for marketing purposes”	Design-questions regarding marketing	Marketing purposes	Pricing vs. social utlity
Yeah, the only thing is I've been in business for 60 years and price has always been a concern (p3, older adult)		price is the concern	Pricing vs. benefit vs. social utlity	Pricing vs. social utlity
So what type of people are you marketing? because one of the things that you brought us with an insurance. Does insurance cover this? and don't get me wrong. P3, older adult; do you think it could be covered by private insurance they cover walkers and wheelchairs but the costs between those two are very different? (clinician)	Insurance coverage	pricing issue	Pricing vs. benefit vs. social utlity	Pricing vs. social utlity
Now you're bringing this unit in at a price that is much higher than the rollator (P3, older adult)	Higher price compared to roller	pricing issue	Pricing vs. benefit vs. social utlity	Pricing vs. social utlity
Do you think it would be covered by private insurance, they cover walkers and wheelchairs but the costs between those two are very different (clinician); Does insuance cover this? (p3, older adult)	Insurance	pricing issue	Pricing vs. benefit vs. social utlity	Pricing vs. social utlity
I like it. but, for three grand I had to cut my tub down to get in and out for shower here and that costed me $2,000. I don't I don't mind spending the money. I think it’s a great piece of equipment. But I think your pricing has to be competitive. And I'm not suggesting that you have to come down to $500. But I think you certainly going to have a marketing problem. Selling that at $3,000. P3, older adult	Competitive pricing	Pricing issues	Pricing vs. benefit vs. social utlity	Pricing vs. social utlity
Price has always been a concern. Now you're bringing this unit in at a price that is much higher than the rollator. So what type of people are you marketing? because one of the things that you brought us with an insurance (older adult)	Unit pricing aligning with the type of people marking	Pricing issues	Pricing vs. benefit vs. social utlity	Pricing vs. social utlity
I like it. but, for three grand I had to cut my tub down to get in and out for shower here and that costed me $2,000. I don't I don't mind spending the money. I think it’s a great piece of equipment. But I think your pricing has to be competitive (p3, older adult)	Competetive pricing	pricing issues	Pricing vs. benefit vs. social utlity	Pricing vs. social utlity
because one of the things that you brought us with an insurance. Does insurance cover this? and don't get me wrong (P3, older adult)	Insurance coverage	pricing issues	Pricing vs. benefit vs. social utlity	Pricing vs. social utlity
I think you need to go to the government and say, this (IBEX lift) has as a social utility that surpasses its utility so to speak as a private device. It saves a lot of money. It saves money and ER vehicles, personnel and so forth. P1, older adult	Safes money, and emergency vehicles and personal	Pricing questions	Pricing vs. benefit vs. social utlity	Pricing vs. social utlity
What is the approximate price? (clinician)	Price	Pricing questions	Pricing vs. benefit vs. social utlity	Pricing vs. social utlity
do you think it could be covered by private insurance they cover walkers and wheelchairs but the costs between those two are very different? (clinician)	Walkers and wheelchairs private insurance coverage vs. Ibexlift coverage	Private insurance coverage	Pricing vs. benefit vs. social utlity	Pricing vs. social utlity
Somebody fell in our parking garage in our building on a Sunday evening. And we called and they said we are really short on ambulances right now. People call in but an hour later, he’s still lying on the floor of the garage. And I finally went out on the street. There are a couple of people coming from the hockey game, strong people and they came to help me get him up (P1, older adult)	Could save cost on ambulance	saving ambulance cost	Pricing vs. benefit vs. social utlity	Pricing vs. social utlity
I think you need to go to the government and say, this (IBEX lift) has as a social utility that surpasses its utility so to speak as a private device. It saves a lot of money. It saves money and ER vehicles, personnel and so forth. (older adult)	Saves money on hospital visits	social ultility	Pricing vs. benefit vs. social utlity	Pricing vs. social utlity
What is the maximum height? So most rollator walkers come in in, you know sort of a low seat, the standard seat and then the tall version. And they do that because the seat heights need to be at different heights. This seat goes from zero to 24 just by design so you can see it can be adjusted and set to any height for any body fat up to 300 pounds. Beyond 300 pounds. It’s not rated for that. (clinician)	Maximum height based on standards	Design-questiions regarding features	Probing Design Question to enhance feature	Probing questions to optimise Ibex lift(c) features and usage
What’s the purpose of the yellow bits? (clinician)	Purpose of device design	Design-question regarding purpose of feature	Probing Design Question to enhance feature	Probing questions to optimise Ibex lift(c) features and usage
Why is the device not movable? Is it because of the seat on the floor? (clinician)	Why the device is stationary during a lift	Design-questiions regarding features	Probing Design Question to enhance feature	Probing questions to optimise Ibex lift(c) features and usage
Will the seat stop lowering if there is an obstacle in the way like someone’s put or cap (clinician)	Obstacle detecting under lift seat	Design-questions regarding features	Probing Design Question to enhance feature	Probing questions to optimise Ibex lift(c) features and usage
When you're like in a position like this, where you're a bit lower, can you propel (clinician); Is it like safe like other later, its not safe to set on and like move around with that. When you say on you have to be still but for that one we can move forward and backwards (clinician)	Shuffling with feet while sitting on the device	Design-question regarding usage	Probing Design Question to enhance usage	Probing questions to optimise Ibex lift(c) features and usage
when you're like in a position like this where you're a bit lower, can you propel? so the answer is sort of it’s not like a wheelchair. The reason is because when you're sitting here so I can move around a little bit but navigating is really hard because you know it looks like they're trying to steer at you have to steer behind because the swivel wheels are out what is now the rear in a wheelchair. (clinician)	Unable to self-propel	Design-question regarding usage	Probing Design Question to enhance usage	Probing questions to optimise Ibex lift(c) features and usage
What do you mean by wrap around the toilet? (clinician)	Using device with the toilet	Design-question regarding use	Probing Design Question to enhance usage	Probing questions to optimise Ibex lift(c) features and usage
What about the battery life if you're there, what does it normal user look like?And what if im somone going up and down, up and down (clinician)	User norm for design	Design-question relating durability	Probing design Question to inquire about durability (Battery)	Probing questions to optimise Ibex lift(c) features and usage
What about the battery if you're there, what does it normal user look like? And what if I'm someone going up and down and up and down? The battery (clinician)	Battery lifetime questioning	Design-questions regarding durability	Probing design Question to inquire about durability (Battery)	Probing questions to optimise Ibex lift(c) features and usage
Is there a battery life indicator somewhere? (clinician)	Importance of batter life indicator	Design-questions regarding durability	Probing design Question to inquire about durability (Battery)	Probing questions to optimise Ibex lift(c) features and usage
Is there a battery life indicator somewhere? (clinician); What about the battery life if you're there, what does it normal user look like?And what if im somone going up and down, up and down	Device Battery	Design-questions relating to durability	Probing design Question to inquire about durability (Battery)	Probing questions to optimise Ibex lift(c) features and usage
What about the battery if you're there, what does it normal user look like? And what if I'm someone going up and down and up and down? so the battery on the new device is going to be embedded in in there permanently and there'll be it'll be sealed. (clinician)	Rechargable battery	Design-questions relating to durability	Probing design Question to inquire about durability (Battery)	Probing questions to optimise Ibex lift(c) features and usage
Is there a battery life indicator somewhere? Yep, right here. Battery indicator is right here and it will flash red when there’s like it's down to less than 20%. So you can still do full down full off. (clinician)	Battery life indicator feature	Design-questions relating to durability	Probing design Question to inquire about durability (Battery)	Probing questions to optimise Ibex lift(c) features and usage
How do you clean the seat if it’s soiled? (clinician)	How to clean seat	Design-questions relating durability	Probing design Question to inquire about durability (How to keep neat)	Probing questions to optimise Ibex lift(c) features and usage
How do you clean the seat if its soiled? (clinician); Are all the parts able to be replaced or purchased of worn out? (clinician); Is there a brake adjustement, because with that type of cable syste, even that they do typically loosen over time and it looks like it’s still got that same type of screw (clinician)	Cleaning and maintanence	design-questions relating to durability	Probing design Question to inquire about durability (How to keep neat)	Probing questions to optimise Ibex lift(c) features and usage
are all the parts able to be replaced or purchased if worn out? (clinician)	Durability of the device	Design-questions relating to durabiliy	Probing design Question to inquire about durability (mainteance and repair)	Probing questions to optimise Ibex lift(c) features and usage
Waterproof casing on it? (clinician)	Does it have water resistance	Design-questions regarding durability	Probing design Question to inquire about durability (waterproof)	Probing questions to optimise Ibex lift(c) features and usage
Is there a brake adjustment because with that type of cable system even that they do typically loosen over time and it looks like it’s still got that same type of screw (clinician)	Durability of the breaks/cable system	Design-questions relating to durabilty	Probing design Question to inquire about durability (maintenace and repair)	Probing questions to optimise Ibex lift(c) features and usage
Is that the max handle height? (clinician)	Max handle Height	Design-questions relating to features	Probing Design Question to enhance feature	Probing questions to optimise Ibex lift(c) features and usage
What is the width of the frame (clinician)	Frame width of device	design-questions relating to features	Probing Design Question to enhance feature	Probing questions to optimise Ibex lift(c) features and usage
What is the width of the frame? It’s 24 inches the final market ready product will be 24 inches. So when you get when the client gets the device, there's two things that need to be set for them just like you would as your fitting rollator walkers for people you adjusted. (clinician)	Customizable width of the seat	Design-questions relating to features	Probing Design Question to enhance feature	Probing questions to optimise Ibex lift(c) features and usage
Although, there’s a problem where you fall in one part of the hotel room and it's (ibex) on another part	Fall away without the IBEX nearby	Design-questions relating to features	Probing Design Question to enhance feature	Probing questions to optimise Ibex lift(c) features and usage
So water resistant, not waterproof, can't take it and shower with it. But it’s water resistant. So if you're out in the rain and the splashes are coming up, you're okay. (clinician)	Water-resistant	Design-questions relating to functional use	Probing design Question to inquire about durability (waterproof)	Probing questions to optimise Ibex lift(c) features and usage
This is getting off the floors is an emergency situation. Have you thought of incorporating an emergency alarm on it? (clinician)	Needs emergency alarm (Suggestion)	Design-suggestions	Design suggestions related to features	Suggestion to optimse Ibex lift (c) features and usage
I'm not suggesting you go shopping in there but it would be nice to have easy access to a cell phone in case you got into trouble and needed to call.	Additional feature promotes usability	Design-suggestions	Design suggestions related to usage	Suggestion to optimse Ibex lift (c) features and usage
If a person is gonna use it outside of the home, a basket on the back, or some type of carrier just for taking some small goods (p1, older adult)	additional features	Design-suggestions	Design suggestions related to usage	Suggestion to optimse Ibex lift (c) features and usage
I'm not suggesting you go shopping in there but it would be nice to have easy access to a cell phone in case you got into trouble and needed to call.P1, older adult	Cell phone carrier	Design-suggestions	Design suggestions related to features and usage	Suggestion to optimse Ibex lift (c) features and usage
.P1If a person is gonna use it outside of the home, a basket on the back, or some type of carrier just for taking some small goods. (informal caregiver)	Basket on the back	Design-suggestions	Design suggestions related to features	Suggestion to optimse Ibex lift (c) features and usage
If a person is gonna use it outside of the home, a basket on the back, or some type of carrier just for taking some small goods. I'm not suggesting you go shopping in there but it would be nice to have easy access to a cell phone in case you got into trouble and needed to call. (older adult)	Suggestions—Adding a basket or carrier	Design-suggestions	Design suggestions related to features	Suggestion to optimse Ibex lift (c) features and usage
If a person is gunna use it outside of the home, a basket on the back, or some type of carrier just for taking some small goods; It would be nice to have easy access to a cell phone in case you got into trouble and needed to call; Is there an accessory basket (clinician)	Basket Attachment	Design-suggestions	Design suggestions related to features	Suggestion to optimse Ibex lift (c) features and usage
If a person is gunna use it outside of the home, a basket on the back, or some type of carrier just for taking some small goods; Can it be used outside (clinician);	Outside usage of device	Design-suggestions	Design suggestions related to features	Suggestion to optimse Ibex lift (c) features and usage
This is getting off the floor in an emergency situation. Have you though of incorporating an energency alarm on it? (clinician)	Emergency Alarm	Design-suggestions	Design suggestions related to features and usage	Suggestion to optimse Ibex lift (c) features and usage
Have you thought of incorporating an emergency alarm on it? (clinician)	Suggestion-addition of emergency alarm to the mobile carriers in case of fall	Design-suggestions	Design suggestions related to features and usage	Suggestion to optimse Ibex lift (c) features and usage
Another thing that can be improved is the weight that was mentioned a couple times and just getting it in and out of the car. (older adult)	Suggestions—Lower the weight for carrying	Design-suggestions	Design suggestions related to features	Suggestion to optimse Ibex lift (c) features and usage
This (backrest) seems to need to be bigger or something. P, older adult	Backrest needs to be bigger	Design-suggestions	Design suggestions related to features	Suggestion to optimse Ibex lift (c) features and usage
Yeah the biggest feature yet (ablity to get off the floor)	Getting off the floor	Design-positive (features)	Design related to features of Ibexlift(c) -	The Synergy of Design-Positive Features in Ibexlift(c) enhancing usage
Like the break button. P2, informal caregiver	Nice break button	Design-positive (features)	Design related to features of Ibexlift(c) -	The Synergy of Design-Positive Features in Ibexlift(c) enhancing usage
So it sounds like the kind of main feature that’s unique and you feel comfortable is the ability to get up off the floor. (older adult)	Positive feedback—Getting up from the floor	design-positives (features)	Design related to features of Ibexlift(c) -	The Synergy of Design-Positive Features in Ibexlift(c) enhancing usage
Another thing that feels good about the weight is that it feelsso much more solid than the lightweight;	Stable and Solid	design-positives (features)	Design related to features of Ibexlift(c) -	The Synergy of Design-Positive Features in Ibexlift(c) enhancing usage
don't I don't mind spending the money. I think it’s a great piece of equipment. (P3, older adult)	Great equipment	Design-positives (general and pricing)	Design related to features of Ibexlift(c) -	The Synergy of Design-Positive Features in Ibexlift(c) enhancing usage
Walker that it would be more helpful than the regular walker. P, older adult	Helpful than a regular walker	Design-positives (usage and general)	Design related to features of Ibexlift(c) -	The Synergy of Design-Positive Features in Ibexlift(c) enhancing usage
Could you lower the price of the thing and make it a fall recovery device and get away and lower its price by reducing other features or is that not a consideration?. P (?)	Use as a fall recovery	Design-positives (usage)	Design related to features of Ibexlift(c) -	The Synergy of Design-Positive Features in Ibexlift(c) enhancing usage
In places where there are no grab bars, which is most places, this would be helpful. I'm not sure of things, grab bars, you know, do prevent falls. But if they're not there or they're in the wrong place, then something like this would be helpful than your regular walker. (informal caregiver)	Positive feedback—Prevent falls instead of grab bars	Design-positives (usage)	Design related to features of Ibexlift(c) -	The Synergy of Design-Positive Features in Ibexlift(c) enhancing usage
Yeah the biggest feature yet (ablity to get off the floor)	Getting off the floor	Design-postive (usage)	Design related to features of Ibexlift(c) -	The Synergy of Design-Positive Features in Ibexlift(c) enhancing usage
P1. Somebody fell in our parking garage in our building on a Sunday evening. And we called and they said we are really short on ambulances right now. People call in but an hour later, he’s still lying on the floor of the garage. And I finally went out on the street. There are a couple of people coming from the hockey game, strong people and they came to help me get him up. (informal caregiver)	OA independence	Experience relating to importance		
